# Novel methodology to discern predictors of remission and patterns of disease activity over time using rheumatoid arthritis clinical trials data

**DOI:** 10.1136/rmdopen-2018-000721

**Published:** 2018-10-25

**Authors:** RA-MAP Consortium

**Keywords:** DAS28 trajectories, latent class mixed models, methotrexate, randomised controlled trial, remission, rheumatoid arthritis

## Abstract

**Objectives:**

To identify predictors of remission and disease activity patterns in patients with rheumatoid arthritis (RA) using individual participant data (IPD) from clinical trials.

**Methods:**

Phase II and III clinical trials completed between 2002 and 2012 were identified by systematic literature review and contact with UK market authorisation holders. Anonymised baseline and follow-up IPD from *non-biological arms* were amalgamated. Multiple imputation was used to handle missing outcome and covariate information. Random effects logistic regression was used to identify predictors of remission, measured by the Disease Activity Score 28 (DAS28) at 6 months. Novel latent class mixed models characterised DAS28 over time.

**Results:**

IPD of 3290 participants from 18 trials were included. Of these participants, 92% received methotrexate (MTX). Remission rates were estimated at 8.4%(95%CI 7.4%to9.5%) overall, 17%(95%CI 14.8%to19.4%) for MTX-naïve patients with early RA and 3.2% (95% CI 2.4% to 4.3%) for those with prior MTX exposure at entry. In prior MTX-exposed patients, lower baseline DAS28 and MTX reinitiation were associated with remission. In MTX-naïve patients, being young, white, male, with better functional and mental health, lower baseline DAS28 and receiving concomitant glucocorticoids were associated with remission. Three DAS28 trajectory subpopulations were identified in MTX-naïve and MTX-exposed patients. A number of variables were associated with subpopulation membership and DAS28 levels within subpopulations.

**Conclusions:**

Predictors of remission differed between MTX-naïve and prior MTX-exposed patients at entry. Latent class mixed models supported differential non-biological therapy response, with three distinct trajectories observed in both MTX-naïve and MTX-exposed patients. Findings should be useful when designing future RA trials and interpreting results of biomarker studies.

**Key messages**

**What is already known about this subject?**

- Clinical remission is achieved in only a minority of patients with rheumatoid arthritis and sustained drug-free remission remains rare. Additionally, response to treatment varies in rheumatoid arthritis.

**What does this study add?**

- Through industry-academic collaboration, individual patient-level data on 3290 patients from the non-biological arms of 18 trials were collated and resulted in the identification of predictors of remission and longitudinal disease activity patterns.
- Differential effects of physical/functional and mental well-being on 6-month Disease Activity Score 28 remission were seen between methotrexate-naïve patients with early disease and those with established disease and prior methotrexate exposure at entry.
- Through novel latent class methodology, three longitudinal patterns of disease activity were discerned in both the baseline methotrexate-naïve and methotrexate-exposed rheumatoid arthritis patient groups.

**How might this impact on clinical practice?**

- Latent class methodology allows both prediction of trajectory membership and future disease course using outcome and covariate information, and can inform trial selection and patient management.

## Background

Rheumatoid arthritis (RA), an inflammatory disease of synovial joints, leads to functional disability and reduced quality of life. Currently, there is no cure but many studies confirm the benefit of early and intensive treatment on long-term outcome.^1 2^ Nonetheless, clinical remission is achieved in only a minority of patients^3 4^ and sustained drug-free remission remains rare.^5 6^

Response to treatment varies in RA. Clinical trials report average disease activity change, but within treatment arms there is heterogeneity; some patients entering clinical remission and some failing to respond. Background disease activity also fluctuates, with some patients demonstrating an initial short-term improvement then either relapsing or plateauing with still relatively active disease irrespective of treatment. Moreover, conventional synthetic disease-modifying antirheumatic drugs (csDMARD) have slow onset of action. Given that prolonged periods of uncontrolled disease activity lead to joint damage and disability, a major unmet need is to identify patient-level predictors of response in order to identify patients with differing patterns of response over time (ie, types of patients with a greater or lesser chance of responding). Such information could guide treatment choices, saving both time and money in achieving sustained disease control; and improve the efficiency of clinical trials.

The Rheumatoid Arthritis (RA-MAP) Consortium is a UK industry-academic partnership funded jointly by the Medical Research Council and the Association of the British Pharmaceutical Industry. RA-MAP’s goal is to investigate clinical and biological predictors of disease outcome in RA, by bringing together experts in basic, clinical, therapeutic development and biostatistical research.^7^ One RA-MAP work stream investigated clinical predictors of remission and response by collation of individual participant data (IPD) from the non-biological arms of randomised controlled clinical trials (RCT). The aims were to identify predictors of response and to identify disease trajectory subpopulations; and then use the findings to inform study design and analysis of future studies.

## Methods

### Identification of relevant studies and study selection

Potential studies were identified by systematic literature review (final search: 13 March 2012) from MEDLINE, EMBASE, PubMed, Ovid, Web of Science, UK Clinical Research Network Portfolio Database (http://public.ukcrn.org.uk/), ClinicalTrials.gov (https://clinicaltrials.gov) and the National Research Register. Searches combined MeSH terms for RA, study type (eg, ‘randomised controlled trial’) and biological and non-biological DMARDs. Additionally, chief investigators of known academic-led clinical trials completed between 2002 and 2012, involving UK patients, were contacted. Current UK market authorisation holders for non-biological and biological DMARDs were also sent a survey via their RA-MAP representative to identify additional trials and seek information on availability of IPD from clinical trials coordinated in the UK or which enrolled UK subjects.

Assessment of trials’ eligibility for inclusion was performed independently by the study coordinator and principal investigator (DS). Lack of consensus was resolved through discussion with the trial’s chief investigator(s), industry sponsor or referral to study publications.

A second literature search was conducted to identify known predictors of remission in RA. This informed the request for baseline data items.

Owners of suitable trial data sets were approached via the RA-MAP representative for access to data on requested variables for all (or a random 80% of) participants in non-biologic arm(s) of these trials. The inclusion criteria, trials obtained and data requested are detailed in online supplementary material. Eligibility of data sets relied on the original informed consent allowing data sharing.

10.1136/rmdopen-2018-000721.supp1 Supplementary data

### Data collection, management and harmonisation

Deidentified data were transferred to the coordinating centre, and further anonymisation added through generation of new unique study identifiers.

Data received were checked for internal consistency, with queries referred back to data owner/supplier. Data were harmonised across trials (ie, given single variable name, standardisation of unit measurement, similar coding of variables when possible) to a standard format for incorporation into a central database. The end product was a pooled database of IPD from trials received. Although a common set of items was requested, some trials, by design, had not collected all items, or when collected, differed in form/construct or level of detail.

### Derived disease activity measure and remission definition

Where possible Disease Activity Score 28 (DAS28) was derived using the four individual components of erythrocyte sedimentation rate (mm/hour), patient global assessment of disease activity (0–100mm visual analogue scale (VAS)) and 28-tender and 28-swollen joint counts.^8^ If patient global assessment was not supplied as a VAS, the three-component DAS28 was calculated.^8^ If direct derivation of DAS28 was not possible then supplied DAS28 was used or the transformation of van Gestel *et al* 9 applied to convert original DAS^10 11^ to DAS28. Clinical remission was defined as DAS28<2.6.^12^

### Sample size evaluation

Initial sample size calculation considered a remission model with 25 significant effects. For simplicity, it assumed that these effects arose from continuous variables that remained statistically significant when dichotomised. A clinically worthwhile detectable difference in remission rates between two groups, formed by median dichotomisation of any predictor, was taken as 4% (eg, 6% remission rate for group below median vs 10% for group above median; giving an overall remission rate of 8%). Assuming a significance level of 0.2% (accounting for multiple testing), a total sample size of 4218 or 2942 is calculated for 95% or 80% power, respectively.

The above scenario was conservative because (1) fewer significant effects could be expected; (2) dichotomisation results in efficiency losses; (3) a 4% difference was considered small; and (4) strict significance level of 0.2% was chosen. It was anticipated that sample sizes above 2500 would be sufficient to achieve the work stream’s aims.

### Statistical methods

The main analyses were based on coprimary outcomes of clinical remission at 6 months (within a 22–26-week window) and DAS28 measured longitudinally. One trial, with 12-week follow-up, was excluded from analyses of remission at 6 months but included in analyses of DAS28 over time. Clinical remission was estimated overall, and separately for methotrexate-naïve (MTX-naïve) entry subjects and those with prior MTX exposure (MTX-exposed). The MTX-exposed group consisted of those on background MTX at trial entry and those who had discontinued MTX.

To identify predictors of remission, (multilevel) random effects logistic regression models (with trial-level random effects) stratified by baseline MTX exposure were considered. If heterogeneity across trials was insubstantial then trial-level random effects were removed. The base model focused on known predictors of remission and potential confounders with limited missing information.^13–15^ It considered the effect of age, sex, ethnicity, disease duration, DAS28, rheumatoid factor (RF) status and RA medication (both prior exposure and as part of study treatment protocol) at baseline. Baseline DAS28 and history of RA medication were also included to adjust for differences in the trial populations due to differing inclusion criteria. Screening models considered separate effects of other potential baseline predictors introduced into the base model. Multivariate models were then built using variables identified as important at screen and forward selection.

Longitudinal latent class mixed models, stratified by MTX exposure at baseline, were used to (1) characterise DAS28 over time (restricted to 1-year follow-up), (2) adjust for potential predictors, (3) incorporate within-patient correlation, and (4) identify cluster trajectories of clinically important subpopulations.^16^ Fixed and random patient-level intercepts, linear and quadratic effects were considered for linear mixed models fitted within latent classes. These random effects were nested within trial. Trial-level random effects were considered, but removed when found inconsequential. (Relative) entropy was calculated to assess the ability of each model to classify individuals into latent classes.^17^ Higher values of entropy indicate better classification of individuals.

Sporadically and systematically missing baseline covariate and missing outcome information at *attended* visits were imputed using multivariate imputation by chained equations,^18 19^ under the missing at random assumption. The hierarchical/multilevel structure (ie, visits within patient, patients within trial) was respected where possible. Twenty imputed data sets were created, analysed and results pooled using Rubin’s rules.^20^

All statistical analyses were performed in R statistical software.^21^ R packages *lme4*,^22^ *mice* 23 and *lcmm* 24 were used for the various analyses.

## Results

### Systematic search and inventory of trials survey

We identified 63 trials to include in the inventory (online supplementary table 1). Sixty trials were industry sponsored (54 from RA-MAP partners) and three academic (from RA-MAP partners). Partial or complete information from study sponsors or publicly available sources was collated for 54/63 trials. There were 8778 patients in non-biological arms of these 54 trials with estimated 6-month remission rate of 8.2%. This estimate informed study sample size (see the Methods section).

### Trials received

Patient-level data from non-biological arms of 19 trials were provided by six industry and two academic RA-MAP partners (see online supplementary table 1). One trial was excluded as it recruited patients with early inflammatory polyarthritis. Patients in the included trials (all started before 2010) met the 1987 American College of Rheumatology RA classification criteria.^25^ Data for 3290 participants from the combined non-biological arms of these 18 trials were obtained. Patient numbers from these trials ranged from 50 to 467. Non-biological assigned treatments included (1) placebo, (2) MTX or other csDMARD monotherapy, or (3) MTX in combination with another csDMARD and/or with glucocorticoid. Placebo-treated patients received either (1) placebo in addition to background RA medication; (2) placebo alone (with RA medication discontinued prior to trial start); or (3) placebo alone (with no prior RA medication; ie, RA medication naïve). Further information on planned duration of RCT phase, inadequate response to csDMARDs, biological intervention, and primary and secondary efficacy outcomes related to disease activity are reported in online supplementary table 1. No data on patients treated in the biological arms of these trials were requested or received.

### Patient characteristics

The baseline demographic and disease characteristics of included patients are summarised in table 1. Only three trials provided information on anti-citrullinated protein antibody status. The mean baseline DAS28 (with SD) was 6.5(1.1).

**Table 1:** Baseline characteristics of patients in 18 trials, overall (n=3290) and stratified by methotrexate (MTX) status prior to randomisation (ie, MTX-naive (n=1137) and MTX-exposed (n=2148); in five patients MTX status was unknown)

Characteristics	Number of trials with information	Overall (n=3290)		MTX-naïve (n=1137)		MTX-exposed (n=2148)	
		Value	% Miss	Value	% Miss	Value	% Miss
Mean age (SD), years	18	52.6 (12.6)	0	52.7 (13.1)	0	52.6 (12.4)	0
Female, %	18	79	0	73.6	0	81.8	0
White, %	18	85.7	0.9	89.3	0.09	83.8	1.3
Rheumatoid factor positive, %	18	75.3	1.8	76.3	3.5	76.6	0.6
Median (IQR) disease duration, years	17	4 (1–10)	5.6	0.67 (0–1.5)	1.8	7 (3–13)	7.7
Mean (SD) age at onset, years	18	46 (13.6)	5.6	50.6 (13.5)	1.8	43.5 (13.0)	7.7
Smoking status, %	10		39.3		57.2		29.7
Non-smoker		14.9		26.5		11.1	
Current smoker		19.2		22.4		18.2	
Not current/ex-smoker		65.9		51.1		70.7	
Mean (SD) 28-tender joint counts	16	15.1 (7.1)	9.6	13.6 (7.6)	14.2	15.8 (6.8)	7.2
Mean (SD) 28-swollen joint counts	16	12.3 (6)	9.6	11.4 (6.3)	14.2	12.8 (5.7)	7.2
Mean (SD) erythrocyte sedimentation rate (ESR), mm/hour	18	46.2 (27)	0.5	44.1 (27.7)	0.18	47.4 (26.5)	0.7
Mean (SD) C-reactive protein (CRP), mg/dL	17	2.64 (3.3)	16.3	3.285 (3.399)	44.1	2.44 (3.24)	1.6
Mean (SD) DAS28 (using ESR)	18	6.5 (1.1)	1.7	6.25 (1.20)	1.1	6.613 (0.939)	2.0
Mean (SD) HAQ	12	1.578 (0.640)	21.3	1.588 (0.663)	2.6	1.571 (0.622)	31.1
Mean (SD) SF-36 Physical Summary Score	12	30.73 (7.73)	24.4	29.95 (8.10)	17.9	31.2 (7.5)	27.7
Mean (SD) SF-36 Mental Summary Score	12	41 (12.31)	24.4	40.84 (13.33)	17.9	41.1 (11.7)	27.7
MTX history status, %	18		0.2				0
MTX-naïve		34.6				0	
On background MTX (ongoing)		54.2				82.9	
Previous MTX use (stopped)		11.2				17.1	
Randomised to or on MTX at start, %*	18	92.1	0	93.1	0	91.8	0
Randomised to or on csDMARD (other than MTX) at start, %*	18	11.9	0	25.2	0	5	0
Randomised to glucocorticoids at start, %*	18	7	0	20.3	0	0	0
Randomised to or on glucocorticoids at start, %*	18	27.4	0	28.4	0	27	0

MTX-exposed (n=2148); in five patients MTX status was unknown.

*Not mutually exclusive categories as patients can be randomised to or receive dual therapy.

csDMARD, conventional synthetic disease-modifying antirheumatic drug; DAS28, Disease Activity Score 28; HAQ, Health Assessment Questionnaire.

Fifty-four per cent of patients were on background MTX at start, 35% were MTX-naïve and 11% had prior MTX exposure (MTX discontinued). Ninety-two per cent of participants were either randomised to MTX or were on background MTX that continued. Twelve per cent was randomised to or continued other csDMARDs. The corresponding percentage for glucocorticoids was 27%. The majority of MTX-naïve patients at entry (93%) were randomised to MTX. Fifty-two per cent of those who discontinued MTX were randomised to MTX reinitiation. A majority of them were viewed as having already demonstrated lack of adequate MTX response.

The 1137 patients who were MTX-naïve at trial entry had substantially shorter median symptom duration than the 2148 patients with prior MTX exposure (8 months vs 7 years, p<0.0001); confirming the fact that the former corresponded to those with early RA.

### Clinical remission at 6 months

Overall 6-month remission rate was estimated at 9.6%(95%CI 8.4%to10.9%) based on 2275 patients for whom 6-month remission could be defined from observed data. After multiple imputation, a 6-month remission rate of 8.4%(95%CI 7.4%to9.5%) was estimated based on 2766 patients who had attended visits within the 22–26week window period. For MTX-naïve entry participants, observed 6-month remission rate was 17.7%(95%CI 15.4%to20.2%) and estimated remission rate after imputation was 17%(95%CI 14.8%to19.4%) based on 1048 patients. For MTX-exposed patients, corresponding estimates were 3.5%(95%CI 2.6%to4.6%) and 3.2%(95%CI 2.4%to4.3%) based on observed data and imputed data from 1718 patients. The adjusted OR of achieving 6-month remission for MTX-exposed versus MTX-naïve patients was 0.26(95%CI 0.17to0.40). Adjustments were made for variables included in the base logistic regression model.

### Predictors of clinical remission at 6 months

#### MTX-naïve at entry

The base (multilevel) random effects logistic regression model for MTX-naïve entry patients is shown in online supplementary table 1. Age, sex, ethnicity, baseline DAS28 and randomised to concomitant glucocorticoids were associated with remission. After screening, three additional variables were considered in building the model further. These were functional disability (Health Assessment Questionnaire, HAQ), SF-36 Physical and Mental Summary Scores.

As HAQ was negatively correlated with SF-36 Physical Summary Score (Pearson correlation of −0.57), two final models (A and B; see table 2) were derived, in which either HAQ or SF-36 Physical Summary Score (but not both together), alongside the SF-36 Mental Summary Score, was considered for inclusion using forward selection. In these models, remission was predicted by being white, male, younger, randomised to concomitant glucocorticoids, having better functional/physical and mental health and lower DAS28, at baseline. Being randomised to concomitant glucocorticoids increased the odds of achieving remission by 4.0(95%CI 2.3to7.2) over not receiving glucocorticoids (model B), controlling for other variables. As most MTX-naïve entry subjects (93%) received MTX, an effect for receiving MTX during the trial could not be estimated, although it was adjusted for in the analysis.

**Table 2:** Final logistic regression model A (including SF-36 summary scores to base model) and model B (including HAQ to base model) for clinical remission at 6months for MTX-naive subjects at entry

Predictors	log(OR)	SE of log(OR)	OR	95% CI for OR	p-value
Final Model A (inclusion of SF-36 summary components to base model)					
Intercept*	–	–	–	–	–
Age at Entry, years	−0.0249	0.0076	0.98	0.96 to 0.99	0.0010
Disease Duration, years	−0.0033	0.0300	1.00	0.94 to 1.06	0.9125
Gender Male v Female	0.9793	0.1953	2.66	1.82 to 3.90	<0.0001
Ethnicity White v Rest	1.3489	0.4957	3.85	1.46 to 10.2	0.0065
DAS28-ESR at Baseline	−0.3616	0.0891	0.70	0.58 to 0.83	<0.0001
Rheumatoid Factor Positivity Yes v No	−0.1352	0.2016	0.87	0.59 to 1.30	0.5024
Randomised to MTX at start* Yes v No	–	–	–	–	–
Randomised to or on csDMARD at start Yes v No	0.1809	0.2726	1.20	0.70 to 2.04	0.5070
Randomised to Glucocorticoids at start Yes v No	1.3375	0.2926	3.81	2.15 to 6.76	<0.0001
On Background Glucocorticoids at start Yes v No	0.2478	0.4857	1.28	0.49 to 3.32	0.6099
SF-36 Physical Summary Score	0.0423	0.0118	1.04	1.02 to 1.07	0.0003
SF-36 Mental Summary Score	0.0209	0.0076	1.02	1.01 to 1.04	0.0063
Final Model B (Inclusion of HAQ to base model)					
Intercept*	–	–	–	–	–
Age at Entry, years	−0.0191	0.0075	0.98	0.97 to 1.00	0.0109
Disease Duration, years	−0.0032	0.0299	1.00	0.94 to 1.06	0.9157
Gender Male v Female	0.8551	0.1945	2.35	1.61 to 3.44	<0.0001
Ethnicity White v Rest	1.3756	0.4937	3.96	1.50 to 10.4	0.0053
DAS28-ESR at Baseline	−0.3489	0.0904	0.71	0.59 to 0.84	0.0001
Rheumatoid Factor Positivity Yes v No	−0.1352	0.2008	0.87	0.59 to 1.29	0.5009
Randomised to MTX at start* Yes v No	–	–	–	–	–
Randomised or on csDMARD at start Yes v No	0.1789	0.2714	1.20	0.70 to 2.04	0.5097
Randomised to Glucocorticoids at start Yes v No	1.3976	0.2920	4.05	2.28 to 7.17	<0.0001
On Background Glucocorticoids at start Yes v No	0.3778	0.4829	1.46	0.57 to 3.76	0.4340
HAQ	−0.6325	0.1616	0.53	0.39 to 0.73	<0.0001

*Estimates and SE are not estimable. MTX usage during study has been adjusted for in models. Majority of MTX-naïve subjects at trial entry received MTX during study (93%).xMark as

csDMARD, conventional synthetic disease-modifying antirheumatic drug; DAS28, DiseaseActivity Score 28; ESR, erythrocyte sedimentation rate; HAQ, Health AssessmentQuestionnaire; MTX, methotrexate.

#### MTX-exposed at entry

The logistic regression (dropping trial-level random effects) in MTX-exposed patients (see table 3) identified lower baseline DAS28 and randomisation to MTX as being associated with achieving 6-month remission. However, patients with prior MTX exposure who were randomised to MTX reinitiation were significantly more likely (p<0.0001) to achieve remission than those continuing on background MTX (adjusted OR 5.2 with 95%CI 2.5 to 10.4). No evidence for functional/physical and mental health effects was found.

**Table 3:** Final logistic regression model for clinical remission at 6months for MTX-exposed subjects

Predictors	log(OR)	SE of log(OR)	OR	95%CI for OR	P values
Intercept	–	–	–	–	–
Age at entry, years	−0.0160	0.0124	0.98	0.96 to 1.01	0.1953
Disease duration, years	−0.0105	0.0206	0.99	0.95 to 1.03	0.6109
Gender: male versus female	0.2935	0.3697	1.34	0.65 to 2.77	0.4271
Ethnicity: white versus rest	−0.0511	0.4137	0.95	0.42 to 2.14	0.9017
DAS28-ESR at baseline	−0.8228	0.1600	0.44	0.32 to 0.60	<0.0001
Rheumatoid factor positivity: yes versus no	−0.5277	0.3214	0.59	0.31 to 1.11	0.1007
MTX use in trial					
(Randomised to MTX, previous use) versus (not receiving, previous use)	1.6499	0.8252	5.21	1.03 to 26.2	0.0456
Background MTX continued versus (not receiving, previous use)	0.0126	0.7874	1.01	0.22 to 4.74	0.9873
Randomised to or on csDMARD at start: yes versus no	1.1721	0.8953	3.23	0.56 to 18.7	0.1905
On background glucocorticoids at start: yes versus no	0.1169	0.3299	1.12	0.59 to 2.15	0.7230

csDMARD, conventional synthetic disease-modifying antirheumatic drug; DAS28, DiseaseActivity Score 28; ESR, erythrocyte sedimentation rate; MTX, methotrexate.

### Characterising disease activity over 1 year of follow-up

Novel latent class mixed modelling of DAS28 suggested the clustering into three subpopulations/classes with differing trajectory profiles in both MTX-naïve and exposed baseline groups. No evidence to support inclusion of trial-level random effects, random slopes or random quadratic effects was found and so the linear mixed models within latent classes contained only fixed effects and random intercepts.

#### MTX-naïve at entry

**Figure 1:**
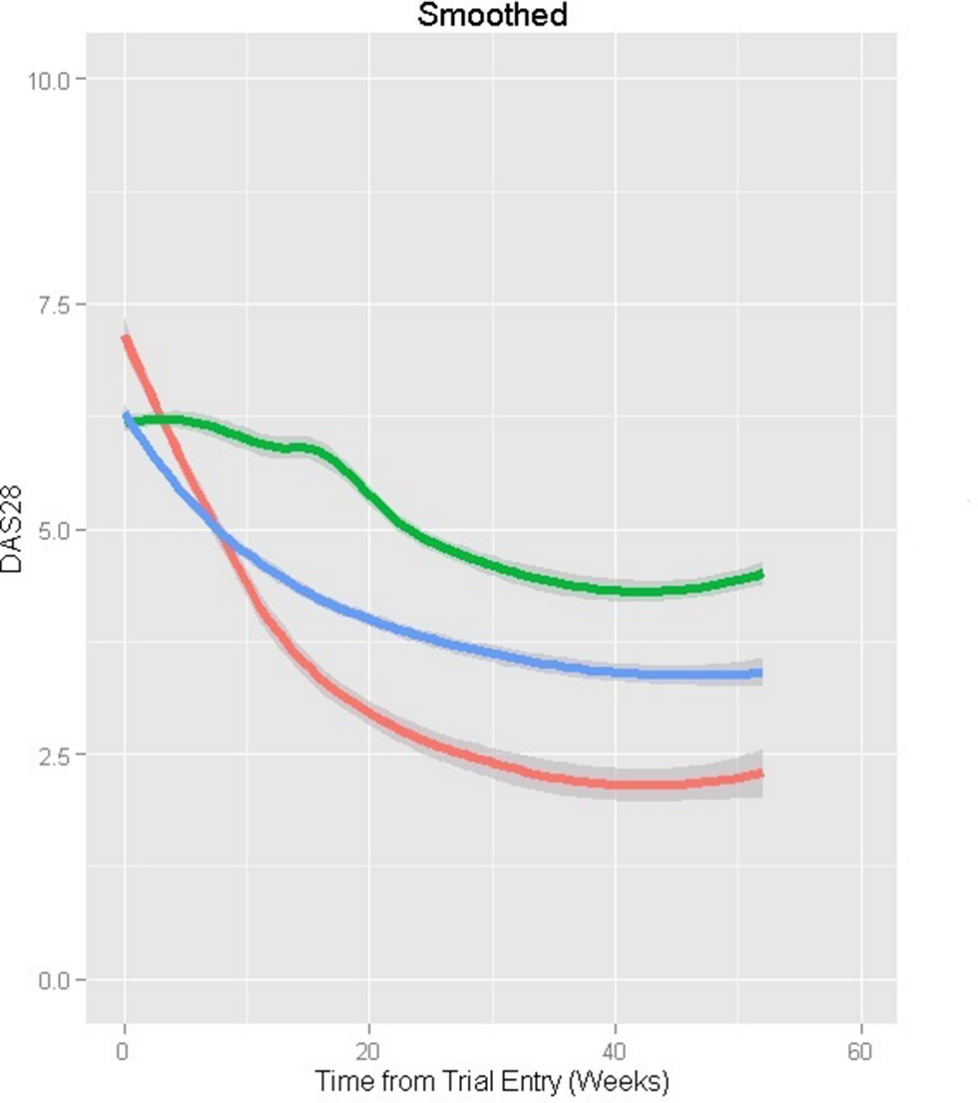
Mean profiles over 1 year from the observed Disease Activity Score 28 (DAS28) data for patients who were methotrexate (MTX)-naïve at trial entry, after stratifying by predicted class membership. Class 1—fast improver group (red): 8%; class 2—moderate improver group (blue): 31.6%; class3—inadequate response group (green): 60.4% (entropy: 0.758).

The three subpopulations identified (table 4 and figure 1; n=1137) corresponded to a fast improver group (class 1; 8% of patients) who, on average, started with higher DAS28; a moderate improver group (class 2; 31.6%) who improved at around half the rate of fast improvers; and an inadequate responder group (class 3; 60.4%) with an improvement rate only 20% of that in class 1. On average, DAS28 of a typical patient with RA would improve by 3.91 in the fast improvers, 2.02 in moderate improvers and 0.56 in inadequate responders over 1 year of follow-up from trial entry.

**Table 4:** Latent class mixed model results for MTX-naïve entry subjects over 1-year follow-up

Predictors	log(OR)	SE	P values
Multinomial class membership model			
Class 1 (fast improver) versus class 2 (moderate improver)			
Intercept	–2.1947	0.4901	<0.0001
Sex: male versus female	0.8881	0.3065	0.0038
Baseline HAQ	0.3416	0.2902	0.2393
Class 3 (inadequate response) versus class 2 (moderate improver)			
Intercept	–1.2339	0.6461	0.0561
Sex: male versus female	0.0128	0.2762	0.9629
Baseline HAQ	0.6174	0.2805	0.0277

Linearmixedmodel	Estimate	SE	P values
Intercept			
Class 1	7.788	0.9264	<0.0001
Class 2	5.6199	0.3158	<0.0001
Class 3	5.7350	0.3400	<0.0001
Disease duration, years			
Class 1	–0.0083	0.0397	0.8335
Class 2	–0.0016	0.0104	0.8768
Class 3	0.0312	0.0126	0.0136
Ethnicity			
White versus rest: class 1	–0.5313	0.3882	0.1711
White versus rest: class 2	–0.2726	0.1837	0.1379
White versus rest: class 3	–0.4331	0.1719	0.0118
Follow-up time in study, weeks			
Class 1	–0.2051	0.0221	<0.0001
Class 2	–0.1117	0.0080	<0.0001
Class 3	–0.0420	0.0080	<0.0001
Follow-up time squared			
Class 1	0.0025	0.0004	<0.0001
Class 2	0.0014	0.0001	<0.0001
Class 3	0.0006	0.0002	<0.0001
Randomised to MTX at start			
Yes versus no: class 1	–1.2085	0.8352	0.1479
Yes versus no: class 2	–0.1143	0.2230	0.6082
Yes versus no: class 3	–0.1299	0.2181	0.5513
Randomised or on csDMARD at start			
Yes versus no: class 1	–0.0597	0.4436	0.8930
Yes versus no: class 2	–0.1673	0.1304	0.1993
Yes versus no: class 3	–0.1127	0.1303	0.3871
Randomised to glucocorticoids at start			
Yes versus no: class 1	–0.3899	0.3976	0.3267
Yes versus no: class 2	–0.3962	0.1480	0.0074
Yes versus no: class 3	–0.2540	0.1550	0.1013
On background glucocorticoid at start			
Yes versus no: class 1	–0.4879	0.3389	0.1500
Yes versus no: class 2	0.2907	0.2262	0.1987
Yes versus no: class 3	0.3468	0.2123	0.1024
HAQ (time varying)			
Class 1	0.4913	0.1045	<0.0001
Class 2	0.5564	0.0698	<0.0001
Class 3	0.6114	0.1035	<0.0001
Variance components*			
Variance of random intercept	0.6308	0.0506	<0.0001
Error SD	0.7941	0.0122	<0.0001
Relative entropy^†^	0.758		

*Trial-levels random effects were investigated and found to be not necessary

†A relative entropy takes values between 0 and 1, with 1 indicating perfect classification

Higher baseline HAQ was associated with having inadequate response. Men were more likely than women to be fast improvers compared with moderate improvers. Higher DAS28 over time in inadequate responders was associated with longer symptom duration (p=0.0136), non-white (p=0.0118) and higher HAQ over time (p<0.0001). In moderate improvers, not being randomised to glucocorticoids (p=0.0074) and higher HAQ over time (p<0.0001) were associated with higher DAS28. In fast improvers, only higher HAQ was associated with higher DAS28 (p<0.0001). The model’s entropy was 0.758, demonstrating good classification. A four-latent class model with the same variables gave lower entropy (0.711).

#### MTX-exposed at entry

**Figure 2:**
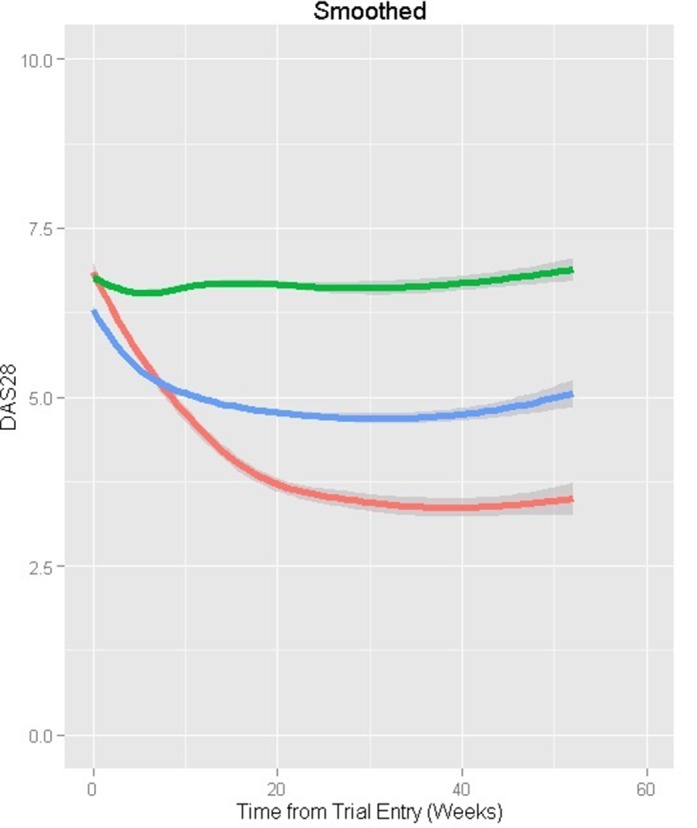
Mean profiles over 1 year from the observed Disease Activity Score 28 (DAS28) data for the methotrexate (MTX)-exposed patients after stratifying by predicted class membership. Class 1—fast improver group (red): 9.4%; class 2—moderate improver group (blue): 43.4%; class 3—inadequate response group (green): 47.3% (entropy: 0.609).

The three subpopulations identified (table 5; figure 2; n=2148) corresponded to a fast improver group (class 1; 9.4% of patients), although not as fast as the corresponding MTX-naïve subpopulation; a group that showed initial improvement but then plateaued and slowly worsened (class 2; 43.4%); and a refractory group (class 3; 47.3%). On average, DAS28 of a typical patient with RA would improve by 3 in the fast improvers, 0.7 in the plateauing group and would worsen by 0.11 in those refractory over 1 year of follow-up.

**Table 5:** Latent class mixed model results for MTX-exposed subjects over 1-year follow-up

Predictors	log(OR)	SE	P values
**Multinomial** **c** **lass** **m** **embership** **m** **odel**			
Class 1 (fast improver) versus class 2 (plateaued)			
Intercept	−1.7852	0.3187	<0.0001
Baseline HAQ	0.4137	0.2073	0.0460
Class 3 (refractory) versus class 2 (plateaued)			
Intercept	−0.7352	0.2987	0.0138
Baseline HAQ	0.5536	0.1648	0.0008

Linearmixedmodel	Estimate	SE	P values
Intercept			
Class 1	5.2433	0.6394	<0.0001
Class 2	5.7507	0.2552	<0.0001
Class 3	5.8371	0.2018	<0.0001
Ethnicity			
White versus rest: class 1	–0.1746	0.1929	0.3655
White versus rest: class 2	–0.2380	0.1034	0.0213
White versus rest: class 3	–0.1233	0.0931	0.1852
Follow-up time in study, weeks			
Class 1	–0.1668	0.0091	<0.0001
Class 2	–0.0863	0.0059	<0.0001
Class 3	–0.0030	0.0042	0.4697
Follow-up time squared			
Class 1	0.0021	0.0001	<0.0001
Class 2	0.0014	0.0001	<0.0001
Class 3	0.0001	0.0001	0.1708
MTX use			
(Randomised MTX, previous on) versus previous on but not randomised: class 1	1.0513	0.6129	0.0863
(Randomised MTX, previous on) versus previous on, but not randomised: class 2	0.0186	0.2750	0.9461
(Randomised MTX, previous on) versus previous on, but not randomised: class 3	0.3206	0.2381	0.1781
Background MTX continued versus previous on but not randomised: class 1	0.9683	0.6140	0.1148
Background MTX continued versus previous on but not randomised: class 2	0.0762	0.2430	0.7539
Background MTX continued versus previous on but not randomised: class 3	0.5088	0.1729	0.0032
Randomised or on csDMARD at start			
Yes versus no: class 1	0.5336	0.6840	0.4353
Yes versus no: class 2	–0.1657	0.3004	0.5813
Yes versus no: class 3	0.6739	0.2151	0.0017
On background glucocorticoids at start			
Yes versus no: class 1	–0.1578	0.1551	0.3088
Yes versus no: class 2	0.0648	0.0843	0.4419
Yes versus no: class 3	–0.1037	0.0788	0.1885
HAQ (time varying)			
Class 1	0.3958	0.0601	<0.0001
Class 2	0.3773	0.0413	<0.0001
Class 3	0.2641	0.0313	<0.0001
Variance components*			
Variance of random intercept	0.6174	0.0269	<0.0001
Error SD	0.6902	0.0056	<0.0001
Relative entropy†	0.609		

*Trial-level random effects were investigated and found to be not necessary.

†A relative entropy takes values between 0 and 1, with 1 indicating perfect classification.

csDMARD, conventional synthetic disease-modifying antirheumatic drug; HAQ, HealthAssessment Questionnaire; MTX, methotrexate.

The ‘plateauing’ group tended to include, on average, patients with lower baseline functional disability. Moreover, in this group, there was evidence to suggest that higher DAS28 associated with being non-white (p=0.0213). Worsening DAS28 was associated with worsening functional disability over time, irrespective of subpopulation. In the refractory group, continuation of background MTX or receiving other csDMARDs as an initial treatment at trial entry was, on average, associated with increased disease activity (DAS28 increase of 0.51, p=0.0032 and 0.67, p=0.0017 respectively) over time.

The model’s entropy was 0.609, demonstrating modest classification. A four-latent class model identified an additional group (around 3.3% of patients) that showed rapid improvement over 3 months and then rebounded dramatically. Although this model had increased entropy (0.659), given the fourth group’s size and unusual pattern, the three-latent class model was preferred.

#### Model outputs

The models presented in tables 4 and 5 are useful for characterising disease activity over time into more homogeneous subpopulations and for identifying predictors of subpopulation membership and disease activity level. The models are also useful for calculating and updating the probabilities of a patient belonging to each of the subpopulations given their current value of DAS28 and covariates and estimated parameters from the model (including estimated random patient-level effects).^24^ This would be particularly useful in an adaptive trial as a new individual recruited could be assigned probabilities of belonging to each trajectory by a model that included all previously recruited individuals.

Furthermore, such models also allow subject-specific predictions of future DAS28 values for patients either given a particular trajectory subpopulation or averaged over all possible trajectory subpopulations. They would also allow population-averaged inference for particular subgroups of patients defined by the values of baseline covariates to inform, for example, national treatment guidelines for patients with RA.

## Discussion

By means of a large industry-academic partnership, IPD from non-biological arms of 18 RA RCTs were amalgamated. These data on 3290 patients allowed a more definitive investigation into clinical predictors of remission, beyond a systematic literature review, through flexible multivariate modelling and novel subgroup analyses using latent class mixed modelling methodology.

We did not aim to do an IPD meta-analysis in order to estimate a common treatment effect across multiple trials investigating the same treatment against the same control intervention. Instead, our goal was to treat this IPD study as an observational cohort in order to more comprehensively investigate the predictors of remission on a variety of non-biological treatments and to discover clinically meaningful subpopulations of patients with RA that could inform the future recruitment of RA patient types into trials and more stratified patient management.

Although patients in RCTs are generally considered to be poorly representative of those patients seen in the general RA clinic population (having higher levels of disease activity at entry and fewer and less severe comorbidities), they nevertheless represent a subpopulation of patients with RA with very real clinical need. Additionally, they represent a patient subpopulation in which treatment management decisions would be made based on the patients’ arthritis symptoms and signs and not complicated by comorbidities and the potential for interactions between the assigned treatments and the comorbidities.

We conducted separate analyses for MTX-naïve and MTX-exposed strata at trial entry, reflecting relatively early and more established disease, respectively. Unsurprisingly, the 6-month remission rate for MTX-naïve (majority then randomised to MTX) patients was substantially higher (17% vs 3.5%) than for those with prior MTX exposure. Utilisation of a treat-to-target strategy, as is usual in clinical practice, may have increased the remission rate in this group further.

A major unmet need is identifying which patients with RA are more or less likely to achieve remission. Our results suggest that, in MTX-naïve entry patients with relatively early disease and high disease activity, baseline factors including age, gender, ethnicity, disease activity, mental health and physical functioning may help identify those with a higher or lower chance of achieving 6-month remission. While all these factors have been previously identified,^14 26 27^ we have confirmed them in a very large sample with the benefits of controlled trial conditions, not usually achievable with large observational studies. These factors should be considered stratifiers when designing future clinical trials and interpreting results of biomarker studies. The potential role of mental health is of current interest, although mechanisms are uncertain, complex and bidirectional.^26 28 29^ The fact that mental and physical/functional well-being was predictive in MTX-naïve entry patients with relatively early disease but not in the prior MTX-exposed entry patients with more established disease is of note and should be explored further as it is difficult in our study to disentangle early/established disease from no/previous exposure to MTX. Some previous studies have shown an effect of smoking status on disease activity^14 30^ we could not confirm this. However, our finding could be due to the high proportion of systematically missing smoking data (57%). RF was not associated with remission here. Previous studies show conflicting results.^14 31 32^

We identified three distinct disease activity trajectories in both MTX-naïve entry and MTX-exposed strata. Although we have given trajectory classes similar names in both strata, the degree of improvement differed depending on MTX exposure history (or early vs established disease at entry through confounding). Siemons *et al* 33 also observed three distinct trajectories from an *unadjusted* latent class mixed model analysis over the first year in patients with early RA.^33^ All their patients followed a treat-to-target strategy and 82% belonged to a ‘fast response’ group with only 3% in a ‘poor response’ group. They found evidence for differences across groups in baseline disease activity measures, pain and SF-36 Physical and Mental Health Summary Scores.^33^ However, they found weaker evidence to support a role of gender in distinguishing groups. We found gender and baseline functional disability were predictors of trajectory class in the MTX-naïve group. The latter was the lone predictor of class membership for MTX-exposed patients. However, the findings of Siemons *et al* were based on one-way analyses of variance rather than introducing variables into their latent class model. There has been debate on whether or not the incorporation of covariates may play an important role in enumerating classes.^34^

A number of variables were associated with DAS28 levels within trajectory classes. In both MTX-naïve and MTX-exposed patients, higher HAQ was associated with higher DAS28 in all classes. Interestingly, in the refractory class of MTX-exposed patients, those who continued background MTX or took other csDMARDs at trial start had higher DAS28 over time. In the class which plateaued, non-whites had higher DAS28 over time. Furthermore, non-whites had higher DAS28 within both moderate improver and inadequate response trajectory classes of the MTX-naïve stratum.

It may seem somewhat confusing that lower disease activity at baseline was associated with achieving clinical remission at 6 months in both baseline MTX-naïve and MTX-exposed groups, and yet there was a subpopulation of baseline MTX-naïve patients who improved rapidly but started with, on average, higher levels of disease activity at baseline than the other two subpopulations of MTX-naïve patients. However, when comparing two patients who differ at baseline with regard to only disease activity (with all other baseline covariates being the same), it is not surprising that the one with the lower baseline disease activity has a higher chance of attaining remission, presumably because he/she has less far to go to attain remission. By comparison, the MTX-naïve subpopulation of ‘fast improvers’ who started with the highest levels of disease activity and rapidly improved, differed from the other MTX-naïve subpopulations in terms of its gender and HAQ baseline distributions. That is, the ‘fast improvers’ had a higher proportion of men and, on average, had higher HAQ values than the other subgroups. Therefore, this ‘fast improvers’ group starts off with higher levels of disease activity and rapidly improves compared with the others primarily because it was made up of patients with a different profile of covariate values in terms of gender and HAQ. It is known that high levels of HAQ at baseline correlate positively with high levels of DAS28 at baseline and that men are more likely to achieve clinical remission at 6 months than women in the MTX-naïve subpopulation (table 2).

When interest is focused on early treatment response and its predictors, then approaches which restrict the longitudinal disease activity response to this early time period rather than the whole follow-up period may be more appropriate. Such approaches would be much more applicable to recent clinical trials in early disease in which aggressive treatment reflects the window of opportunity and treat-to-target goals.

There are many advantages of combining data from multiple trials. However, one methodological challenge is data harmonisation across trials; in particular here, with regard to creating a common DAS28 variable. There is ongoing debate as to the exact equivalence of DAS28 calculated using different formulae and the validity of combining different methods of calculation in the same analysis. These issues could impact on findings, although we believe less so in characterising disease activity over time. There is also debate over the extent to which DAS28 remission cut-off overestimates remission, as defined by absence of residual inflammatory disease activity.^35^ However, DAS28 remission remains a widely used and aspirational target in clinical practice and trials, and we do not believe this invalidates our findings.

Even though our analyses were done using relatively large sample sizes, there is still the need to validate the findings before these results/models could be used to inform clinical practice or trial selection. There is a potential for our models to be overoptimistic due to the model fitting process and multiple testing. In addition, models which incorporate routinely collected biomarkers may have more clinical utility.

The existence of differing trajectories supports a stratified medicine approach and suggests the potential for tailoring treatments to distinct patient subpopulations. Moreover, trajectories and predictors of response may differ by drug class. For example, these latent class mixed models would allow us, using the disease activity measure at screening (or past disease activity measures) and baseline covariate information, to estimate the likely trajectory pattern an MTX-naïve patient with high disease activity would take if they were to enter a trial and be randomised to a non-biological arm. If it was important, in this trial, to select only patients who had a high probability of responding to treatment, then our models could identify those patients who were least likely to respond (ie, the inadequate responders) to the control treatment and exclude them from the trial. These types of models could also be used in clinical practice (when validated), for example, to assign a probability of response to different choices or combinations of csDMARDs by an MTX-naïve patient with active disease (assuming that the clinician had access to their past disease activity values and covariate information). The prediction of trajectory class or the likely response to a change in treatment could be refined at follow-up visits using current disease activity values.

The entropies of our models, for both MTX-naïve and MTX-exposed strata, show room for improvement in classification accuracy. We anticipate that the addition of novel immune biomarkers, being investigated by the RA-MAP Consortium through their inception cohort study, will lead to predictor models that are clinically informative when choosing treatments for patients with RA.

10.1136/rmdopen-2018-000721.supp2 Supplementary data

10.1136/rmdopen-2018-000721.supp3 Supplementary data
