# Supplementary material for: Novel methodology to discern predictors of remission and patterns of disease activity over time using rheumatoid arthritis clinical trials data
Source: RMD Open. 2018 Oct 25;4(2):e000721. doi: 10.1136/rmdopen-2018-000721 (PMC6241979; doi:10.1136/rmdopen-2018-000721)
Supplement: Supplementary data [file rmdopen-2018-000721supp001.docx]

**Supplementary Material**

Inclusion criteria:

1. RCT of either conventional synthetic (including glucocorticoids) or biologic disease modifying anti-rheumatic drugs (DMARDs) in early inflammatory polyarthritis, or RA
2. RCT co-ordinated in the UK or included enrolment of UK-based participants and been completed between 2002 and 2012 (to increase chance of access)
3. Phase II or III RCT of three or more months treatment duration
4. RCT must include 80% or more of the required individual-level and trial-level information
5. Participants aged 16 years and over
6. Original informed consent must have permitted the sharing of data
7. Sponsors agree to share de-identified/anonymised patient level data

The information requested comprised

1. Trial information: inclusion/exclusion criteria and treatment regimes;
2. Randomisation details;
3. Baseline data: demographics (e.g. age, sex, smoking), clinical (e.g. disease duration, rheumatoid factor (RF) and anti-citrullinated protein antibody (ACPA) status, disease activity measures including erythrocyte sedimentation rate (ESR) and/or C-reactive protein (CRP) , radiographic damage), patient reported outcome measures (PROMs) (e.g. Health Assessment Questionnaire (HAQ) score; Short Form 36 Health Survey (SF-36), Fatigue) and medication history;
4. Follow-up data: the timing of follow-up visits, end of study status, reasons for withdrawal, disease outcome information including disease activity variables (28-joint counts, acute-phase reactants, global assessment of disease activity, disease activity scores etc.), radiographic damage, and PROMs, co-medications and treatment received, and treatment departure and adherence.


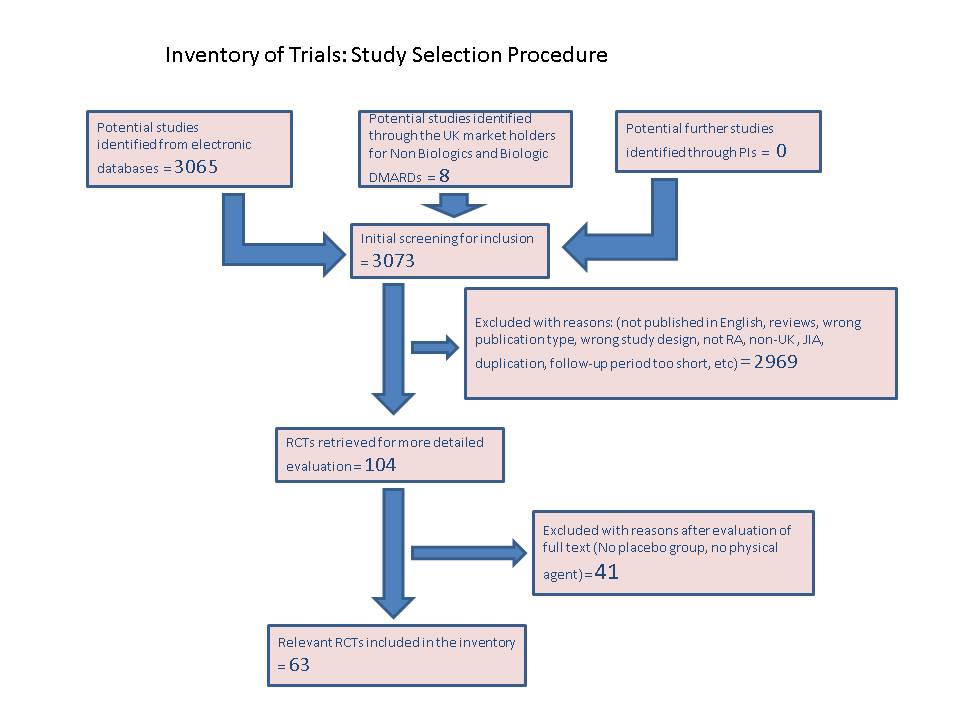


**Supplementary Figure 1: Flow diagram summarising the study selection procedure**

**Supplementary Table 1a: Description of the 19 Randomised Controlled Trials received (Stivea trial excluded from all analyses)**

| **Trial Identifier**  **(Start year)** | **Trial design** | **Planned duration of RCT phase (weeks)** | **Control arm treatment(s)** | **Open label extension part?** | **Failed to respond to at least one csDMARD**  **as an inclusion criteria** | **Did some patients have prior MTX use?** | **Number of patients reported in control arm(s)** | **Number of patients extracted from control arm(s)** |
| --- | --- | --- | --- | --- | --- | --- | --- | --- |
| NCT00291928  (2005) | Phase I/II | 48 | Placebo | No | Yes | Yes | 63 | 63 |
| NCT00713544  (2008) | Phase IIb | 12 | Placebo + Background MTX | No | Yes | Yes | 65 | 65 |
| NCT00548834*  (2003) | Phase III multi-centre  (UK site?) | 24 | Placebo | Yes** | Yes | Yes, but discontinued | 109 | 88 |
| NCT00152386*  (2005) | Phase III  multi-centre | 52 | Placebo + BackgroundMTX | Yes** | Yes^#^ | Yes | 199 | 162 |
| ETANERCEPT STUDY 309  (Before 2006) | Phase III  multi-centre | 24 | Sulfasalazine (SSZ) | No | Yes^##^ | Yes, but discontinued | 50 | 50 |
| NCT00195494  (2004) | Phase III  multi-centre | 52 | MTX | No | No | No | 268 | 268 |
| NCT00393471  (2000) | Phase III  multi-centre | 52 | MTX | No | Yes^###^ | Yes, but discontinued | 228 | 231^^^ |
| STIVEA***  (2002) | Phase III  multi-centre | 52 | Placebo and IM Glucorticoid | No | No | No | 265 | 265 |
| CARDERA  (2000) | Phase III multi-centre factorial trial | 104 | MTX, MTX + Ciclosporin, MTX + Prednisone and Triple Therapy | No | No | No | 467 | 467 |
| NCT00544154*  (2002) | Phase III multi-centre | 24 | Placebo + Background MTX | Yes** | Yes^#^ | Yes | 121 | 98 |
| NCT00611455  (2008) | Phase III multi-centre | 24 | Placebo + Background MTX | Yes** | Yes^#^ | Yes | 131 | 134^^^ |
| NCT00111423~  (2001) | Phase II | 24 | Placebo + Background MTX | No | Yes^#^ | Yes | 77 | 77 |
| NCT00037700  (2001) | Phase II | 24 | Placebo + Background MTX | No | Yes^#^ | Yes | 68 | 68 |
| NCT00106522****  (2005) | Phase III multi-centre | 24 | Placebo + Background MTX | No | Yes | Yes | 160 | 160 |
| NCT00106535****  (2005) | Phase III multi-centre | 52^@^ | Placebo + Background MTX | Yes^@@^ | Yes | Yes | 393 | 392^^^^ |
| NCT00106548****  (2005) | Phase III multi-centre | 24 | Placebo + Background MTX | No | Yes | Yes | 204 | 204 |
| NCT00109408  (2005) | Phase III multi-centre | 24 | MTX | No | No | Yes, but discontinued | 284 | 284 |
| NCT00106574****  (2005) | Phase III multi-centre | 24 | Placebo + Background DMARD therapy | Yes** | Yes | Yes | 415 | 414^^^^ |
| NCT00520572  (2007) | Phase IIb | 28 | Placebo + Background MTX or SSZ | No | Yes | Yes | 65 | 65 |

* Random 80%; ** Encouraged or eligible to enter an open-label extension study if withdrawn or after completion (open-label data not available); *** Study of patients with very early inflammatory polyarthritis (excluded from analyses); ^#^ Active disease despite on MTX; ^##^ Active disease despite on SSZ; ^###^ Not MTX; ^^^ Includes patients who although randomised did not receive test article or randomised in error; **** Rescue therapy offered at 16/8 weeks in all cases of treatment failure (41.25%, 50%, 33.33%, 3.9% and 10.8% resp.) ; ^@^ Only have 24 weeks of data; ^@@^ Second year of open-label therapy and an optional 3-year open-label extension phase; ^^^^ Patients who actually received the control treatment; ~The Trial identifier corresponds to the extension study to the original trial which does not have an NCT identifier.

**Supplementary Table 1b: Description of the 19 Randomised Controlled Trials received (Stivea Trial excluded from all analyses)**

| **Trial Identifier**  **(Start year)** | **Trial design** | **Biological Intervention** | **Primary Efficacy Outcome**  **(disease activity-related)** | **Secondary Efficacy Outcomes**  **(disease activity-related)** |
| --- | --- | --- | --- | --- |
| NCT00291928  (2005) | Phase I/II | Ofatumumab | ACR20 and DAS28 over time  (ACR20 at 24 weeks) | EULAR response  (EULAR at 24 weeks) |
| NCT00713544  (2008) | Phase IIb | AZD5672 | ACR20 at 12 weeks | ACR50/70 at 12 weeks; Change in DAS28 after 12 weeks |
| NCT00548834  (2003) | Phase III multi-centre  (UK site?) | CDP870 | ACR20 at 24 weeks | ACR50/70 at 24 weeks; ACR component scores; DAS28(ESR) 3 |
| NCT00152386  (2005) | Phase III  multi-centre | CDP870 | ACR20 at 24 weeks | ACR20 at 52 weeks; ACR50/70 at 24 and 52 weeks |
| ETANERCEPT STUDY 309  (Before 2006) | Phase III  multi-centre | ETANERCEPT | ACR20 at 24 weeks | ACR20/50/70, DAS and its components over time |
| NCT00195494  (2004) | Phase III  multi-centre | ETANERCEPT | DAS28 remission at 52 weeks; | DAS44 remission, ACR20/50/70 |
| NCT00393471  (2000) | Phase III  multi-centre | ETANERCEPT | ACR response AUC over 24 weeks | ACR20/50/70 and DAS |
| STIVEA  (2002) | Phase III  multi-centre | None | Need to start DMARDs by 6 months | DAS28(3) |
| CARDERA  (2000) | Phase III multi-centre factorial trial | None | None | Changes in DAS28 |
| NCT00544154  (2002) | Phase III multi-centre | CDP870 | ACR20 at 24 weeks | ACR50/70 at 24 weeks; ACR component scores |
| NCT00611455  (2008) | Phase III multi-centre | Ofatumumab | ACR20 at 24 weeks | ACR50/70, EULAR response and DAS28 at 24 weeks |
| NCT00111423  (2001) | Phase II | Pegsunercept | ACR20 at 24 weeks | Sustained ACR20/50/70 response at 24 weeks, DAS, change from baseline in ACR component scores |
| NCT00037700  (2001) | Phase II | Anakinra, Pegsunercept | No details available from ClinicalTrials.gov | No details available from ClinicalTrials.gov |
| NCT00106522  (2005) | Phase III multi-centre | Tocilizumab | ACR20 at 24 weeks | ACR50/70 at 24 weeks; Changes in ACR component scores; DAS28 and EULAR response |
| NCT00106535  (2005) | Phase III multi-centre | Tocilizumab | ACR20 at 24 weeks | ACR20/50/70; DAS28 |
| NCT00106548  (2005) | Phase III multi-centre | Tocilizumab | None | ACR50/70 at 24 weeks; Change from baseline in DAS28 at 24 weeks; DAS28 remission and EULAR response |
| NCT00109408  (2005) | Phase III multi-centre | Tocilizumab | ACR20 at 24 weeks | ACR20 at 8 weeks; ACR50/70 at 24 weeks; Change in ACR components; DAS28 and EULAR response |
| NCT00106574  (2005) | Phase III multi-centre | Tocilizumab | ACR20 at 24 weeks | ACR20 at 8 weeks; ACR50/70 at 24 weeks; Change in ACR components; DAS28; EULAR response and Time to ACR20/50/70 response |
| NCT00520572  (2007) | Phase IIb | AZD9056 | ACR20 at 24 weeks | ACR50/70; Changes in ACR components; DAS28 |

**Supplementary Table 2: Base logistic regression model for clinical remission at 6 months for MTX-naïve entry subjects**

| Predictors | log(Odds Ratio) | Standard Error | Odds Ratio | 95% CI | p-value |
| --- | --- | --- | --- | --- | --- |
| Intercept* | ---- | ---- | ---- | ---- | ---- |
| Age at Entry, years | -0.0188 | 0.0074 | 0.98 | 0.97-1.00 | 0.0109 |
| Disease Duration, years | -0.0111 | 0.0294 | 0.99 | 0.93-1.05 | 0.7062 |
| Gender  Male v Female | 0.9761 | 0.1914 | 2.65 | 1.82-3.86 | <0.0001 |
| Ethnicity  White v Rest | 1.3524 | 0.4886 | 3.87 | 1.48-10.1 | 0.0056 |
| DAS28-ESR at Baseline | -0.5028 | 0.0825 | 0.60 | 0.51-0.71 | <0.0001 |
| Rheumatoid Factor Positivity  Yes v No | -0.1460 | 0.1990 | 0.86 | 0.59-1.28 | 0.4630 |
| Randomised to MTX at start*  Yes v No | ---- | ---- | ---- | ---- | ---- |
| Randomised to or on csDMARD at start  Yes v No | 0.1594 | 0.2696 | 1.17 | 0.69-1.99 | 0.5543 |
| Randomised to Glucocorticoids at start  Yes v No | 1.3558 | 0.2905 | 3.88 | 2.20-6.86 | <0.0001 |
| On Background Glucocorticoids at start  Yes v No | 0.2638 | 0.4795 | 1.30 | 0.51-3.33 | 0.5823 |

* Estimates and standard error are not estimable. MTX usage during study has been adjusted for in models. Majority of MTX-naïve subjects at trial entry received MTX during study (93%).
