# Supplementary material for: Novel methodology to discern predictors of remission and patterns of disease activity over time using rheumatoid arthritis clinical trials data
Source: RMD Open. 2018 Oct 25;4(2):e000721. doi: 10.1136/rmdopen-2018-000721 (PMC6241979; doi:10.1136/rmdopen-2018-000721)
Supplement: Supplementary data [file rmdopen-2018-000721supp002.pdf]

Paper title
**Novel methodology to discern predictors of remission and patterns of disease activity over time using rheumatoid arthritis clinical trials data**
**Author**
**RA-MAP Consortium**
**Corresponding Authors**
**Brian Tom and Deborah Symmons**

List of Contributing Authors (for REF purposes only) – in alphabetical order

Name	Affiliation	Please provide details of contribution	Competing interest statement (if any)
Sarah Brockbank	Institute of Cellular Medicine, Newcastle University	RA-MAP project manager: Overall management and facilitate the process of data acquisition and analysis	None
Claudio Carini	Inflammation and Immunology RU<br>Worldwide Research & Development Pfizer Inc.	Provision of strategic input and patient level data; critical review of manuscript	None
Andrew P. Cope	Academic Department of Rheumatology, Division of Immunology, Infection and Inflammatory Disease, Faculty of Life Sciences, King's College London	Workpackage Lead for this study: contributions to study set up, acquisition and provision of datasets and analysis, and critical review of the manuscript	None
Michael R Ehrenstein	Division of Medicine, UCL	Contributed to concept and design of study; interpretation of the data; critical review of the manuscript	None
Benjamin A Fisher	University of Birmingham and University Hospitals Birmingham NHS Trust and Sandwell and West Birmingham Hospitals NHS Trust	Contributed to analysis and interpretation of data, and drafting and critical review of the manuscript.	None
Carl S. Goodyear	Institute of Infection, Immunity and Inflammation, College of Medical, Veterinary and Life Sciences, University of Glasgow, Glasgow, UK	Contributed to concept and design of study; interpretation of the data; critical review of the manuscript	Research funding, consultancy and/or honoraria from Abbvie, AstraZeneca, Becton Dickenson, BMS, Janssen, MedImmune, and Pfizer
Neil Gozzard	UCB Pharma	Provision of strategic input and patient level data; critical review of manuscript	None
Ray Harris	Eisai Limited<br>European Knowledge Centre<br>Mosquito Way<br>Hatfield<br>Herts	Provision of advisory statistical input throughout the project and review of the statistical methodology	An employee of Eisai Limited.

	AL10 9SN	used in the paper.	
Kirsty Hicks	Statistics and Programming, Pharma R&D, GlaxoSmithKline	Contributed to study design; statistical analyses of data; critical review of the manuscript	Shareholder in GSK
Sally Hollis	Biometrics and Information Sciences, Global Medicines Development, AstraZeneca	Contributed to statistical analyses of data; critical review of the manuscript	None
Adwoa Hughes-Morley	Centre for Musculoskeletal Research, School of Biological Sciences, Faculty of Biology, Medicine and Health, University of Manchester<br>NIHR Manchester Musculoskeletal Biomedical Research Unit, Central Manchester NHS Foundation Trust, Manchester Academic Health Science Centre	Contributed to the acquisition, analysis and interpretation of data; critical review of the manuscript	None
John Isaacs	NIHR Newcastle Biomedical Research Centre in Ageing and Long-term Conditions, Institute of Cellular Medicine, Newcastle University and Musculoskeletal Unit, Newcastle upon Tyne Hospitals NHS Foundation Trust	RA-MAP Consortium Academic Lead: contribution to the design of the study; critical review and approval of manuscript	Consultant to: Pfizer, Lilly, Abbvie, Janssen, Celltrion, Epirus, Boehringer, Baxalta<br>Research support: Pfizer<br>Speaker: Roche, BMS, Pfizer
Blerina Kola	Medical Director Inflammation, Pfizer Ltd, Walton Oaks, Walton on the Hill, Tadworth, Surrey, KT20 7NS, U.K	Collection and integration of patient level data; critical review of manuscript	Pfizer employee and Pfizer shareholder
Iain B. McInnes	Institute of Infection, Immunity and Inflammation, College of Medical, Veterinary and Life Sciences, University of Glasgow, Glasgow, UK	Contribution to the study concept; critical revision of manuscript	Grants and/or personal fees from AbbVie, AstraZeneca, BMS, Celgene, Crescendo Bioscience, Janssen, MSD, Novartis, Lilly, UCB, Amgen, and Pfizer
Christopher M Mela	Roche Products Ltd. 6 Falcon Way, Shire Park, Welwyn Garden City, AL7 1TW. UK.	Contribution to acquisition, analysis and interpretation of data; critical review of manuscript	CM Mela is an employee of Roche Products Ltd.
Gerry Parker	UCB Pharma	Provision of strategic input and patient level data; critical review of manuscript	None
Ayako Wakatsuki Pedersen	Institute of Cellular Medicine, Newcastle University	RA-MAP project manager: Overall management and facilitate the process of data acquisition and analysis	None
Frederique Ponchel	Leeds Institute of Rheumatic and Musculoskeletal Medicine,	Contribution to the study concept; critical revision	None

	University of Leeds	of manuscript	
Tony Sabin	Medical Sciences Biostatistics group at Amgen	Provision and analysis of patient level data; input to statistical methodology; Contribution to the study concept; critical revision of manuscript	Former Amgen employee
David L. Scott	Academic Department of Rheumatology, Division of Immunology, Infection and Inflammatory Disease, Faculty of Life Sciences, King's College London	Contributed to the data acquisition and critical review of the manuscript	None
Ian C. Scott	Academic Department of Rheumatology, Division of Immunology, Infection and Inflammatory Disease, Faculty of Life Sciences, King's College London	Contributed to the data acquisition and critical review of the manuscript	None
Matthew A. Sleeman	Respiratory, Inflammation & Autoimmunity, MedImmune Ltd, Cambridge, UK.	Contributed to the study design; provision of data interpretation; critical review of the manuscript	M. Sleeman was a full time employee of MedImmune, a wholly owned subsidiary of Astrazeneca.
Deborah Symmons	Centre for Musculoskeletal Research, School of Biological Sciences, Faculty of Biology, Medicine and Health, University of Manchester<br>NIHR Manchester Musculoskeletal Biomedical Research Unit, Central Manchester NHS Foundation Trust, Manchester Academic Health Science Centre	Contributed to the conception and design of the work; drafted and critical revision of the manuscript	None
Peter C. Taylor	Norman Collisson Professor of Musculoskeletal Sciences<br>Head of Clinical Sciences<br>Botnar Research Centre<br>Nuffield Department of Orthopaedics, Rheumatology and Musculoskeletal Sciences, University of Oxford<br>Botnar Research Centre, Windmill Road<br>Headington, Oxford, OX3 7LD.	Contribution to the study concept; critical revision of manuscript	**Research grants to Oxford University**<br>UCB, GSK, Celgene, Galapagos, Abide therapeutics.<br>**Consultation, and/or speaking**<br>Roche, UCB, GSK, BMS, Lilly, Pfizer, Janssen, AbbVie, Biogen, Sandoz, Novartis, Sanofi.
Brian Tom	MRC Biostatistics Unit, University of Cambridge	Design of the work, data acquisition and cleaning, statistical analysis, interpretation and writing; approval of final version of manuscript and accountable for all aspects of the work	None
Wayne Tsuji	Medical Sciences, Early Development , Amgen	Provision of strategic input and patient level data; critical review of	Former Amgen employee; Amgen shareholder

		manuscript	
Yujie Zhong	MRC Biostatistics Unit, University of Cambridge	Statistical analysis, interpretation and revising manuscript critically	None
