# Supplementary material for: Novel methodology to discern predictors of remission and patterns of disease activity over time using rheumatoid arthritis clinical trials data
Source: RMD Open. 2018 Oct 25;4(2):e000721. doi: 10.1136/rmdopen-2018-000721 (PMC6241979; doi:10.1136/rmdopen-2018-000721)
Supplement: Supplementary data [file rmdopen-2018-000721supp003.pdf]

RA-MAP Membership

Academic Members

<u>Newcastle University (Institute of Cellular Medicine)</u>
(<u>John Isaacs:</u> NIHR Newcastle Biomedical Research Centre in Ageing and Long-term Conditions, Institute of Cellular Medicine, Newcastle University and Musculoskeletal Unit, Newcastle upon Tyne Hospitals NHS Foundation Trust)
John Isaacs
Sarah Brockbank
Ayako Wakatsuki Pedersen
Catharien Hilkens
Amy Anderson
Philip Stocks
Dennis Lendrem
Jessica Tarn
Graham Smith
Ben Allen
John Casement
Julie Diboll
Rachel Harry

<u>Kings College London</u>
(1) Academic Department of Rheumatology, Division of Immunology, Infection and Inflammatory Disease, Faculty of Life Sciences, King's College London
Andrew P. Cope
Gemma Simpson
Ruth Toward
Hayley Noble
Angela Parke
Wing Wu
Fiona Clarke
David Scott
Ian C Scott
James Galloway
Heidi Lempp
Fowzia Ibrahim
Samana Schwank
Gemma Molyneux

(2) Department of Immunobiology, Division of Immunology, Infection and Inflammatory Disease, Faculty of Life Sciences, King's College London *(Current address: Memorial Sloan Kettering Cancer Centre, New York, USA)*
Tomi Lazarov
Frederic Geissmann

<u>University of Glasgow</u>
(1) Institute of Infection, Immunity and Inflammation, College of Medical, Veterinary and Life Sciences, University of Glasgow, Glasgow, UK
Carl S. Goodyear
Iain B. McInnes
Iona Donnelly
Ashley Gilmour
Aysin Tulunay Virlan

(2) Gartnavel General Hospital, Glasgow, UK

Duncan Porter

<u>University of Leeds (</u>Leeds Institute of Rheumatic and Musculoskeletal Medicine)
Frederique Ponchel
Paul Emery
Jehan El-Jawhari
Rekha Parmar
Michael F. McDermot
Maya Buch

<u>University of Birmingham</u>
Benjamin A Fisher
Chris Buckley
Steve P Young
Philip Jones
Karim Raza
Andrew Filer
[**BAF, CB and AF:** University of Birmingham and University Hospitals Birmingham NHS Trust and Sandwell and West Birmingham Hospitals NHS Trust]
[**KR**: University of Birmingham and Sandwell and West Birmingham Hospitals NHS Trust]

<u>QMUL</u>
(1) Centre for Experimental Medicine and Rheumatology, William Harvey Research Institute, Barts and The London School of Medicine and Dentistry, Queen Mary University of London , London , UK
(2) Centre for Translational Bioinformatics, William Harvey Research Institute, Barts and The London School of Medicine and Dentistry, Queen Mary University of London , London , UK
Costantino Pitzalis (1)
Michael R Barnes (2)
David S Watson (2)
Evan Tzanis(2)
Georgina Thorborn (1)
Liliane Fossati-Jimack (1)
Stephen Kelly (1)
Frances Humby (1)
Michele Bombardieri (1)
Sharmila Rana (1) & (2)
Zhilong Jia (2)
Katriona Goldmann (1) & (2)
Myles Lewis (1)

<u>University College London (Division of Medicine)</u>
Michael R Ehrenstein
Gioia Altobelli
Chris John
Sandra Martins
Dao Nguyen
Humayara Ali
Coziana Ciurtin

<u>University of Manchester</u> (Centre for Musculoskeletal Research, School of Biological Sciences, Faculty of Biology, Medicine and Health, University of Manchester
NIHR Manchester Biomedical Research Centre, Manchester University Foundation Trust, Manchester Academic Health Science Centre)
Deborah Symmons
Jane Worthington
Ian N Bruce
Jamie C Sergeant

Suzanne MM Verstappen

Fiona Stirling
Adwoa Hughes-Morley (current: York Trials Unit, Department of Health Sciences, Faculty of Sciences University of York, Heslington YO10 5DD)

MRC Biostatistics Unit, University of Cambridge
Brian Tom
Vernon Farewell
Yujie Zhong

University of Oxford (Norman Collisson Professor of Musculoskeletal Sciences
Head of Clinical Sciences, Botnar Research Centre, Nuffield Department of Orthopaedics, Rheumatology and Musculoskeletal Sciences, University of Oxford, Botnar Research Centre, Windmill Road,Headington, Oxford, OX3 7LD)
Peter C. Taylor

Industry Members

Abbvie Inc

(1) Medical Affairs, Abbvie Ltd, Maidenhead SL6 4UB, UK
(2) Translational Immunology, AbbVie Bioresearch Center Inc., Worcester, MA, USA.
(3) Immunology Clinical Development, Abbvie Bioresearch Center Inc., Worcester, MA, USA.
(4) Immunology Pharmacology, AbbVie Bioresearch Center Inc., Worcester, MA, USA.
(5) Information Research, AbbVie Bioresearch Center Inc., Worcester, MA, USA.
(6) Global Biologics, AbbVie Bioresearch Center Inc., Worcester, MA, USA.
(7) Exploratory Statistics, AbbVie Bioresearch Center Inc., Worcester, MA, USA.

Sarah Keidel (1)
Carolyn Cuff (2)
Marc Levesque (3)
Andrew Long (4)
Zheng Liu (2)
Samantha Lipsky (5)
Bohdan Harvey (6)
Michael Macoritto (2)
Feng Hong (7)
Sukru Kaymakcalan (5)

Amgen Inc

Wayne Tsuji (current: Cascadia Drug Development Group)
Tony Sabin (current: Biostatistics & Information Practice Department, AstraZeneca)
Neil Ward
Susan Talbot
Desmond Padhji

MedImmune

Respiratory, Inflammation & Autoimmunity, MedImmune Ltd, Cambridge, UK.

Matthew Sleeman (Current affiliation : Dept Immunology & Inflammation, Regeneron Pharmaceuticals, Tarrytown, NY, USA)
Donna Finch
Athula Herath (Biostatistics, MedImmune Ltd, Cambridge, UK. Current: Novartis Pharmaceuticals UK Ltd, Frimley, Camberley, Surrey GU16 7SR.)

AstraZeneca

Global Medicines Development, Astrazeneca

Catharina Lindholm
Martin Jenkins
Meilien Ho [deceased]
Sally Hollis (current: Statistical Consulting, Phastar)
Chris Marshall (current: BioSci Consulting)

UCB Pharma

Neil Gozzard
Gerry Parker
Matt Page
Hannah Edwards
Alexandru Cuza

Janssen

(1) Janssen Research & Development Ltd, High Wycombe, UK
(2) Janssen Pharmaceutica NV, 2340, Beerse, Belgium

(3) Janssen Research & Development, LLC, Spring House, Pennsylvania, USA

Anthony Rowe (3)
Francisco Bonachela Capdevila (2)
Matthew Loza (3)
Mark Curran (3)
Denny Verbeeck (2)
Dan Baker (3)

Roche
Roche Products Ltd. 6 Falcon Way, Shire Park, Welwyn Garden City, AL7 1TW. UK.

Christopher M Mela
Ivana Vranic
Catherine T Mela
Stephen Wright
Lucy Rowell
Emma Vernon
Nina Joseph
Neil Payne

GSK
(1) Specialty Franchise, Global Medical, Pharma R&D
(2) ImmunoInflammation TA, Pharma R&D
(3) Respiratory TA, Pharma R&D
(4) Statistics and Programming, Pharma R&D
(5) Target Sciences Computational Biology, Pharma R&D
(6) Vaccines, R&D

Ravi Rao (1)
Michael Binks (current: Rare Disease Clinical Research, Pfizer)
Alexandra Belson (2)
Valerie Ludbrook (3)
Kirsty Hicks (4)
Hannah Tipney (5)
Joanne Ellis (2)
Samiul Hasan (5)
Arnaud Didierlaurent (6)
Wivine Burny (6)
Andrea Haynes (2)
Chris Larminie (5)

Eisai Ltd
European Knowledge Centre, Mosquito Way, Hatfield, Herts, AL10 9SN

Ray Harris
Daniela Dastros-Pitel

Pfizer
(1) Inflammation and Immunology RU, Worldwide Research & Development Pfizer Inc.
(2) Medical Director Inflammation and Immunology, International Developed Markets, Pfizer Ltd, Tadworth, Surrey, KT20 7NS, U.K

Claudio Carini (current: Dept of Asthma, Allergy & Lung Biology, KCL)
Blerina Kola (2)
Scott Jelinsky (1)
Martin Hodge (1)
Mateusz Maciejewski (1)
Daniel Ziemek (1)

Protagen
Protagen AG, 44227 Dortmund, Otto-Hahn Street 15, Germany

Peter Schulz-Knappe
Hans-Dieter Zucht
Petra Budde

SimOmics Ltd
Kennedy Institute of Rheumatology, University of Oxford, Roosevelt Drive, Headington, Oxford, OX3 7FY, UK

Mark C. Coles
James A. Butler

Grunenthal
Grunenthal GmbH, Zieglerstraße 6, 52078 Aachen, Germany

Simon Read
